# *In situ* correction of various β-thalassemia mutations in human hematopoietic stem cells

**DOI:** 10.3389/fcell.2023.1276890

**Published:** 2024-01-25

**Authors:** Yinghong Yang, Lina He, Yingjun Xie, Lifen Zhu, Jianfeng Wu, Yong Fan, Yi Yang, Xiaofang Sun

**Affiliations:** ^1^ Guangdong Provincial Key Laboratory of Major Obstetric Diseases, Department of Obstetrics and Gynecology, Guangdong Provincial Clinical Research Center for Obstetrics and Gynecology, Guangdong-Hong Kong-Macao Greater Bay Area Higher Education Joint Laboratory of Maternal-Fetal Medicine, The Third Affiliated Hospital of Guangzhou Medical University, Guangzhou, China; ^2^ Department of Reproductive Medicine, Zigong Hospital of Women and Children Health Care, Guangzhou, China

**Keywords:** β-thalassemia, CRISPR/Cas9, hematopoietic stem cells, recombinant adeno-associated viral 6, gene therapy

## Abstract

β-thalassemia (β-thal) is the most common monogenic disorder caused by various mutations in the human hemoglobin β (HBB) gene and affecting millions of people worldwide. Electroporation of Cas9 and single-guide RNA (sgRNA)–ribonucleoprotein (RNP) complex-mediated gene targeting in patient-derived hematopoietic stem cells (HSCs), followed by autologous transplantation, holds the promise to cure patients lacking a compatible bone marrow donor. In this study, a universal gene correction method was devised to achieve *in situ* correction of most types of *HBB* mutations by using validated CRISPR/sgRNA–RNP complexes and recombinant adeno-associated viral 6 (rAAV6) donor-mediated homology-directed repair (HDR) in HSCs. The gene-edited HSCs exhibited multi-lineage formation abilities, and the expression of β-globin transcripts was restored in differentiated erythroid cells. The method was applied to efficiently correct different mutations in β-thal patient-derived HSCs, and the edited HSCs retained the ability to engraft into the bone marrow of immunodeficient NOD-scid-IL2Rg−/− (NSI) mice. This study provides an efficient and safe approach for targeting HSCs by HDR at the HBB locus, which provides a potential therapeutic approach for treating other types of monogenic diseases in patient-specific HSCs.

## Introduction

β-thalassemia (β-thal) is the most prevalent autosomal recessive blood disorder caused by either point mutations or small deletions of nucleotides in the HBB gene; so far, more than 200 different types of mutations have been reported (Origa, 2017). In erythrocytes, adult hemoglobin (HbA) consists of two α-globin and two β-globin chains; β-thal results in decreased, abnormal, or absent synthesis of the β-globin chain, which generates an imbalance between the subunits of HbA, leading to unpaired α-globin chains precipitating in erythroid cells and causing ineffective erythropoiesis, hemolysis, and varying severity of anemia (Shah et al., 2019). β-thal affects millions of people worldwide, and approximately 1,500 new births each year are affected by a severe form of β-thal (Biffi, 2018). In the most severe form of β-thal, patients require long-term blood transfusions and chelation therapy (Cavazzana et al., 2017), and to date, no effective treatments are available.

The only curative treatment for β-thal is hematopoietic stem cell transplantation. However, it is limited by the lack of human leukocyte antigen (HLA)-identical donors and the risk of graft-versus-host disease (Jagannath et al., 2016). In recent decades, scientists have developed an alternative approach of lentivirus-mediated gene therapy for β-thal on the basis of transducing the normal β-globin gene into the patient’s own hematopoietic stem cells (Thompson et al., 2018). However, the risk of random lentiviral integration and transgene silencing remains a long-term safety concern (Magrin et al., 2019). Recent clinical studies, based on the reactivation of endogenous γ-globin expression by CRISPR/Cas9 targeting the BCL11A erythroid-specific enhancer, are a promising therapeutic strategy for β-thal (Frangoul et al., 2021). However, the lentiviral expression of the β-globin gene or the induction of fetal hemoglobin (HbF) with various approaches leaves the disease-causing mutation alleles intact, resulting in the concentration of primary pathogenic hemoglobin polymerizing in erythroid cells. Currently, alternative precise genome editing strategies have been proposed, and studies have aimed to achieve *in situ* correction of the HBB mutation through HDR, which could activate the endogenous promoters regulating spatiotemporal gene expression of the HBB gene in HSCs (Dever and Porteus, 2017; Antony et al., 2018).

In recent years, CRISPR/Cas9 genome editing technology has been widely applied to disrupting or correcting genetic mutations in cells for clinical gene therapies (Chen et al., 2021; Wilkinson et al., 2021). Although many endonuclease nickase-based HBB correction approaches have been reported, most of the studies focused on a specific mutation, such as the sickle cell disease (SCD) locus in exon 1 of *HBB* (Dever et al., 2016; Hoban et al., 2016). In contrast to SCD, which is caused by a single point mutation in the sixth codon of the HBB gene, β-thal contains more than 200 types of mutations, each of which could be corrected by using a validated guide RNA and the specific donor. Therefore, devising a strategy to correct most types of HBB mutations by using validated guide RNAs and a universal DNA template for future clinical applications is highly desirable. Recombinant adeno-associated viral 6 (rAAV6) has recently emerged as a leading gene transfer system for clinical trials, with sufficient transduction efficiency of human HSCs and low immunogenicity (Wang et al., 2019). In the present study, an HBB gene correction strategy was developed using ribonucleoprotein (RNP) delivery of Cas9 protein and a validated sgRNA targeting intron 2 of *HBB*, followed by delivery of a repairing donor with the use of rAAV6 in HSCs, which could be applicable to most forms of β-thal. Notably, the approach was applied to efficiently correct mutations at different positions in β-thal patient-derived HSCs, and it maintained the engraftment ability when transplanted into NSI mice. Taken together, this study presents a general Cas9/sgRNA-rAAV6-mediated genome-editing platform of HSCs at the HBB locus for the treatment of not only β-thal but also of a wide range of other HSC-based hematological diseases and gene therapies for basic and translational research.

## Materials and methods

### Acquisition and culture of CD34^+^ hematopoietic stem cells

CD34^+^ HSCs from cord blood and peripheral blood were obtained from the Department of Obstetrics and Gynecology at The Third Affiliated Hospital of Guangzhou Medical University. Blood samples from patients with β-thal were obtained from Dongguan Taixin Hospital, and all experiments were performed with the approval of the local medical ethics committee. The HSCs and cord blood HSCs of patients with β-thal were enriched via density gradient separation and CD34^+^ Microbead Kit Ultrapure (130-100-453, Miltenyi Biotec). FITC mouse anti-human CD34 (555821, BD Biosciences) was used for cell staining for CD34 to test their purity. The HSCs were cultured in StemSpan SFEM II (09605, STEMCELL Technologies) supplemented with 100 ng/mL SCF (255-SC-200, R&D Systems), 100 ng/mL TPO (300-18-50, PeproTech), 100 ng/mL Flt3L (300-19-10, PeproTech), 100 ng/mL IL-6 (200-06-50, PeproTech), 0.75 µM StemRegenin1 (72352, STEMCELL Technologies), and 35 nM UM171 (72914, STEMCELL Technologies). The cells were cultured at 37°C and 5% CO_2_.

### Design and production of sgRNAs and AAV6 vector

The sgRNAs targeting intron 2 of *HBB* were designed online (http://crispor.tefor.net/) with the following genomic sgRNA target sequences with bold PAMs: sgRNA1 (5ʹ-ATA​GGA​AGG​GGA​TAA​GTA​AC AGG-3ʹ); sgRNA2 (5ʹ-GTT​AAG​TTC​ATG​TCA​TAG​GA AGG-3ʹ); sgRNA3 (5ʹ-GAC​GAA​TGA​TTG​CAT​CAG​TG TGG-3ʹ); and sgRNA4 (5ʹ-AAC​AGG​GTA​CAG​TTT​AGA​AT GGG-3ʹ). A pair of complementary DNA oligonucleotides were annealed and ligated to the BbsI sites of the PUC57-T7 cloning vector. The recombinant vector was amplified with T7 primers (T7-F: 5′-GAA​ATT​AAT​ACG​ACT​CAC​TAT A-3′ and T7-R: 5′-AAA​AAA​AGC​ACC​GAC​TCG​GTG​CCA​C-3′). The PCR products of gRNA were transcribed using the MEGAshortscript T7 Transcription Kit (AM1354, Life Technologies) and recycled using the RNeasy MinElute Cleanup Kit (74204, QIAGEN) in accordance with the manufacturer’s instructions. Modified sgRNA was purchased from Thermo Fisher Scientific, with chemical modifications at the terminal nucleotides of the 5ʹ and 3ʹ ends.

**FIGURE 1:**
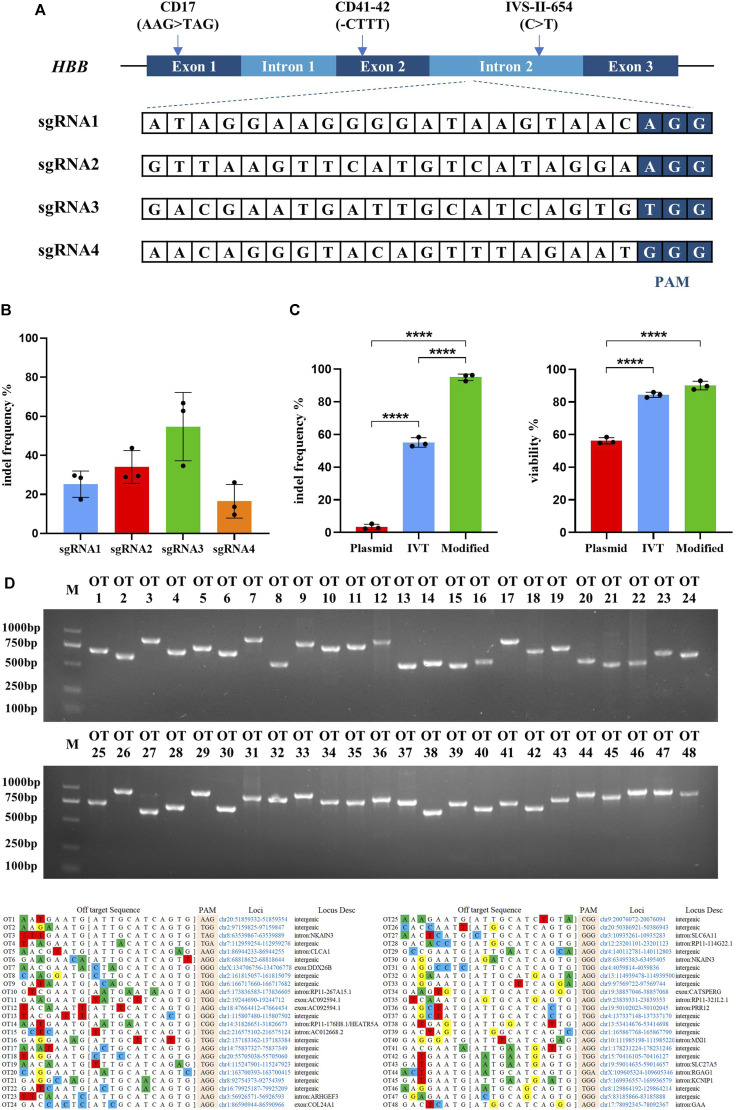
Highly efficient sgRNA screening targeted on *HBB* intron locus and optimizing the delivery of Cas9/sgRNA RNP into hematopoietic stem cells. All experiments in this figure were performed on cord blood-derived CD34^+^ cells. **(A)** Genomic structure of the β-globin gene cluster and schematic of sgRNA targeting sites. Four sgRNAs were designed targeting the intron 2 of the HBB locus. **(B)** Indel frequency detection of synthesized Cas9 mRNA with different sgRNAs electroporated into cord blood-derived HSCs and assessed by TIDE assay. Values are presented as the mean ± SD for triplicate samples from a representative experiment. *p*-values were calculated using one-way ANOVA. ** indicates <0.01. **(C)** Indel frequency and cell viability of different sgRNA treatments in plasmid, *in vitro* transcription (IVT), or chemically modified sgRNA electroporated into HSCs. **(D)** The top 48 potential off-target sites were predicted using the online software CRISPOR. T7E1 assays were conducted to assess mutagenesis at the predicted off-target sites of sgRNA3.

The AAV vector plasmids containing inverted terminal repeats (ITRs) from AAV serotype 2 (AAV2) were purchased from PackGene Biotech. The 4.7 kb AAV packaging capacity limits the size of the donor. So the rAAV6 homologous recombination repair template was designed to contain a 1708 bp arm and a 507 bp arm of the β-globin locus (Figure 1). The donor also contained BGH polyA, EGFP, and SFFV promoters. The rAAV6 virus was purchased from PackGene, and the titer value was determined to be 1E+13 GC/mL by quantitative PCR detection. The prepared rAAV6 virus vector was divided and stored at −80°C for future use.

### Electroporation and transduction of cells

RNP was constructed by incubating Cas9 protein (A36499, Thermo Fisher Scientific) with sgRNA (IVT-sgRNA or modified sgRNA) at a molar ratio of 1:2.5 (Cas9 protein: sgRNA) for 30 min at room temperature before electroporation. The CD34^+^ HSCs were electroporated using the Neon Transfection System (Thermo Fisher Scientific) with the electroporation parameters of 1600 V, 10 ms, and 3 pulses 3 days after thawing or isolation. A 100 µL system was used with the following conditions: 1 × 10^6^ cells, 15 µg Cas9 protein, and 7.5 µg sgRNA. After electroporation, the donor packaged by AAV6 was added immediately into the media of cells at an MOI of 1,000–20,000 (vector genome/cell). An additional co-infection reagent of 8 μg/mL polybrene (C0351, Beyotime) was added, as well as other small-molecule compounds, including 5 µM SCR7 (S7742, Selleck) and 5 µM L755507 (S7974, Selleck). All cells were then incubated overnight at 37 C and 5% CO_2_.

### Measurement of the indel frequency

Genomic DNA was extracted from cells at least 2 days after electroporation to validate the most efficient targeting efficiency of Cas9/sgRNA. The PCR products spanning the Cas9-sgRNA cleavage site were sequenced by Sanger, and TIDE software (http://shinyapps.datacurators.nl/tide/) was used (by disaggregating tracking indels) to quantify the indel frequency. The primers used to amplify the *HBB* fragment and for sequencing were as follows: *HBB*-intron2-indel-F (5ʹ-GAG​TGA​GCT​GCA​CTG​TGA​CAA-3ʹ) and *HBB*-intron2-indel-R (5ʹ-AGA​ATG​GTG​CAA​AGG​CAT​GAT​AC-3ʹ).

### Off-target assay

A total of 48 potential off-target sites of sgRNA3 were predicted using CRISPOR online software (http://crispor.tefor.net/). PCR products (500–1000 bp) containing the potential off-target site were sequenced and confirmed by T7E1 enzyme digestion to identify exact gene mutations.

### Whole-genome sequencing and Sanger sequencing

Genomic DNA was extracted from the WT, Cas9/sgRNA3 RNP + AAV6, and Cas9/sgRNA3 RNP + AAV6-GFP cells by using the Genome Extraction Kit (QIAGEN, 69,504). Whole-exome sequencing was applied to detect the coding exons and untranslated regions, which was performed on the Illumina NovaSeq 6000 Sequencer with a paired-end 2 × 150 nt multiplex. In contrast to the human genome Hg19, the data were analyzed using BWA. A Genome Analysis Toolkit (GATK, version 2.8.1) was used to detect SNVs and indels.

### Methylcellulose colony-forming unit (CFU) assay and genotyping

Colony-forming unit (CFU) assay was performed to evaluate the gene targeting efficiency and the multi-lineage differentiation ability of gene-edited HSCs. Ninety-six hours post-electroporation and transduction with AAV6, the EGFP^+^ high population was sorted by FACS, and approximately 1 × 10^4^ cells were suspended in MethoCult H4434 Classic (04434, STEMCELL Technologies) and placed in 35-mm culture dishes. The cells in the dishes were incubated at 37 C with 5% CO_2_ in the air at 95% humidity. The colonies in the dishes were counted using a microscope and classified as CFU erythroid (CFU-E), burst-forming unit-erythroid (BFU-E), CFU-granulocyte/macrophage (CFU-GM), and CFU-granulocyte/erythroid/macrophage megakaryocyte (CFU-GEMM) in accordance with their morphology. After 14 days, the individual colonies formed in methylcellulose were aspirated, washed with PBS, and centrifuged at 300 *g* for 5 min to precipitate the cells. The cells were then resuspended with 10 µL DNA lysis buffer (1% NP40 plus 550 ng/μL protein K) and transferred to plates for PCR. The detection of integrated or non-integrated alleles by in-out PCR was performed to detect the 3ʹ end EGFP integration rate of *HBB*. The primer sequences were as follows: *HBB*-in-out-PCR-F1 (5ʹ-TCC​CCC​TGA​ACC​TGA​AAC​ATA​AAA​T-3ʹ); *HBB*-in-out-PCR-F2 (5ʹ-TAA​AAA​GGG​AAT​GTG​GGA​GGT​CA-3ʹ); *HBB*-in-out-PCR-R (5ʹ- TTT​GGG​GTG​GGC​CTA​TGA​CA-3ʹ).

### Differentiation of CD34^+^ HSCs into erythrocytes *in vitro*

Erythroid differentiation of CD34^+^ HSCs was performed according to the manufacturer’s instructions (09600, STEMCELL Technologies). In the first phase, corresponding to days 0–10, the cells were cultured at 10^5^ cells/mL in StemSpan SFEM Medium supplemented with 10% FBS (35-081-CV, Corning), 50 ng/mL SCF (255-SC-200, R&D Systems), 10 ng/mL IL-3 (200-03-50, PeproTech), 1 IU/mL EPO (18802B, ESPO), and 100 IU/mL penicillin/streptomycin (15,140-122, Gibco). In the second phase, corresponding to days 11–21, the media were replaced with StemSpan SFEM Medium supplemented with 30% FBS, 3 IU/mL EPO, and 100 IU/mL penicillin/streptomycin. The erythrocyte differentiation was assessed via flow cytometry (Invitrogen Attune NxT, Thermo Fisher Scientific) using the following antibodies: PE mouse anti-human CD71 (555537, BD Biosciences) and APC anti-human CD235a (349114, BioLegend). The erythrocytes were stained by Wright–Giemsa staining (BA4017, BASO) and imaged using a microscope (DMi1, Leica Microsystems).

### Assessment of mRNA levels in differentiated erythrocytes

RNA was extracted from differentiated 14-day-old HSCs by using the RNeasy Micro Kit (74004, QIAGEN) and reversed into cDNA by using the RT Reagent Kit with gDNA Eraser (RR047A, TaKaRa). The mutation sites and repair of cDNA were identified by PCR and Sanger sequencing. The primer sequences used were as follows: *HBB*-cDNA-F (5ʹ-ACA​ACT​GTG​TTC​ACT​AGC​AAC​C-3ʹ) and *HBB*-cDNA-R (5ʹ-AGC​AAG​AAA​GCG​AGC​TTA​GTG-3ʹ). The relative amount of mRNA was determined by fluorescence quantitative PCR (qPCR) using QuantStudio 3 (A31665, Thermo Fisher Scientific). Calculations were performed in accordance with the comparative CT method, and the relative gene expression levels were normalized to those of the housekeeping gene *GAPDH*. The primer sequences used were as follows: *HBB*-qPCR-F (5ʹ-CAC​CTT​TGC​CAC​ACT​GAG​TGA​G-3ʹ); *HBB*-qPCR-R (5ʹ-CCA​CTT​TCT​GAT​AGG​CAG​CCT​G-3ʹ); *GAPDH*-qPCR-F (5ʹ-GTC​TCC​TCT​GAC​TTC​AAC​AGC​G-3ʹ); *GAPDH*-qPCR-R (5ʹ-ACC​ACC​CTG​TTG​CTG​TAG​CCA​A-3ʹ).

### Transplantation of gene-edited HSCs into NSI mice and engraftment ability assay

Immunodeficient NSI mice aged 7–8 weeks old were randomly assigned to each experimental group and used for *in vivo* studies. Four days after electroporation of Cas9 ribonucleoproteins and transduction with rAAV6-mediated HDR in HSCs, 1,000,000 cells were directly administered by tail-vein injection into the NSI mice with sublethal irradiation (200 cGy) using an insulin syringe with a 27 gauge × 1/2 inch needle, respectively. Four months after transplantation, the mice were euthanized, and the cells in the lumen of the bone marrow (BM) chamber were harvested to determine the engraftment potential and the rate of targeted HSCs. Mononuclear cells (MNCs) were enriched by using Ficoll-Paque PLUS (GE Healthcare) by gradient centrifugation at 800 *g* for 25 min. Then, the erythrocytes were lysed with RBC erythrocyte lysate and resuspended using FACS buffer. The monoclonal flow antibody mixture (4°C, protected from light, 30 min) was added, including PerCP-Cy5.5 mouse anti-human CD45 (564105, BD Biosciences), APC/Cyanine 7 anti-human HLA.B.C (311426, BioLegend), PE anti-human CD33 (983904, BioLegend), and APC anti-human CD19 antibody (561742, BD Biosciences). The stained cells were then resuspended in the FACS buffer and analyzed on a BD FACSAria Ⅲ. Human engraftment was determined by HLA-ABC^+^/hCD45^+^ cells. Normal multi-lineage engraftment was determined by the surface markers of B cells (hCD19^+^) and myeloid cells (hCD33^+^). The GFP expression within engrafted human CD45^+^ HLA-ABC^+^ cells was analyzed via flow cytometry, and it represents the percentage of targeted cell engraftment.

### Statistical data

All data were statistically processed using SPSS 19.0 software. Means between multiple groups were compared using one-way ANOVA. The experimental results were presented as mean ± SD. *p* < 0.05 was used as the criterion for statistically significant differences.

## Results

### Design and optimization of CRISPR/Cas9 targeting the HBB locus in HSCs

Identifying the most effective sgRNA to achieve the highest efficiency of Cas9/gRNA-created DSB is a critical factor for the endonuclease-mediated HDR in HSCs. Four gRNAs targeting intron 2 of the human HBB gene were designed and synthesized (Figure 1A). The targeting efficiency of the four gRNAs was validated by transfecting synthesized Cas9 mRNA and gRNAs into the cord blood-derived HSCs. TIDE analysis showed that sgRNA3 was more effective (Figure 1B). Several studies reported that sgRNA chemically modified at both termini with 2ʹ O-methyl 3′phosphorothioate (MS sgRNA) and delivered in conjunction with Cas9 protein as an RNP complex by electroporation is the most effective method for creating DSBs in cells, which is degraded rapidly with reducing off-target effects (Kim et al., 2014; Yin et al., 2017). The efficiency of Cas9/sgRNA3-encoding plasmid, *in vitro*-transcribed Cas9/sgRNA3 mRNA, and Cas9/sgRNA3–RNP complex electroporated into cord blood-derived HSCs were compared, and the use of the Cas9/sgRNA3 RNP complex acquired an average of 96.3% indel efficiency, with minimized cell death (Figure 1C, Supplementary Figures S1A, B). Then, the different electroporation parameters of HSCs were compared, and the results illustrated that 1600 V was the most optimized electroporation parameter for the high indel frequency and cell survival rate (Supplementary Figure S1C). Thus, the delivery method of the Cas9/sgRNA3–RNP complex was optimized for HSCs, resulting in high rates of cleavage at the HBB locus, with minimized cell death. Off-target effect is also a major concern of the CRISPR/Cas9 system. The potential off-target sites (POTs) were computationally predicted to exclude the off-target effects of Cas9/sgRNA3. All POTs were PCR-amplified and subjected to the T7E1 cleavage assay, and the results showed that no mutation was identified in these POTs (Figure 1D), demonstrating the high fidelity of Cas9/sgRNA3-mediated gene editing.

### Development of a universal HBB gene targeting strategy

**FIGURE 2:**
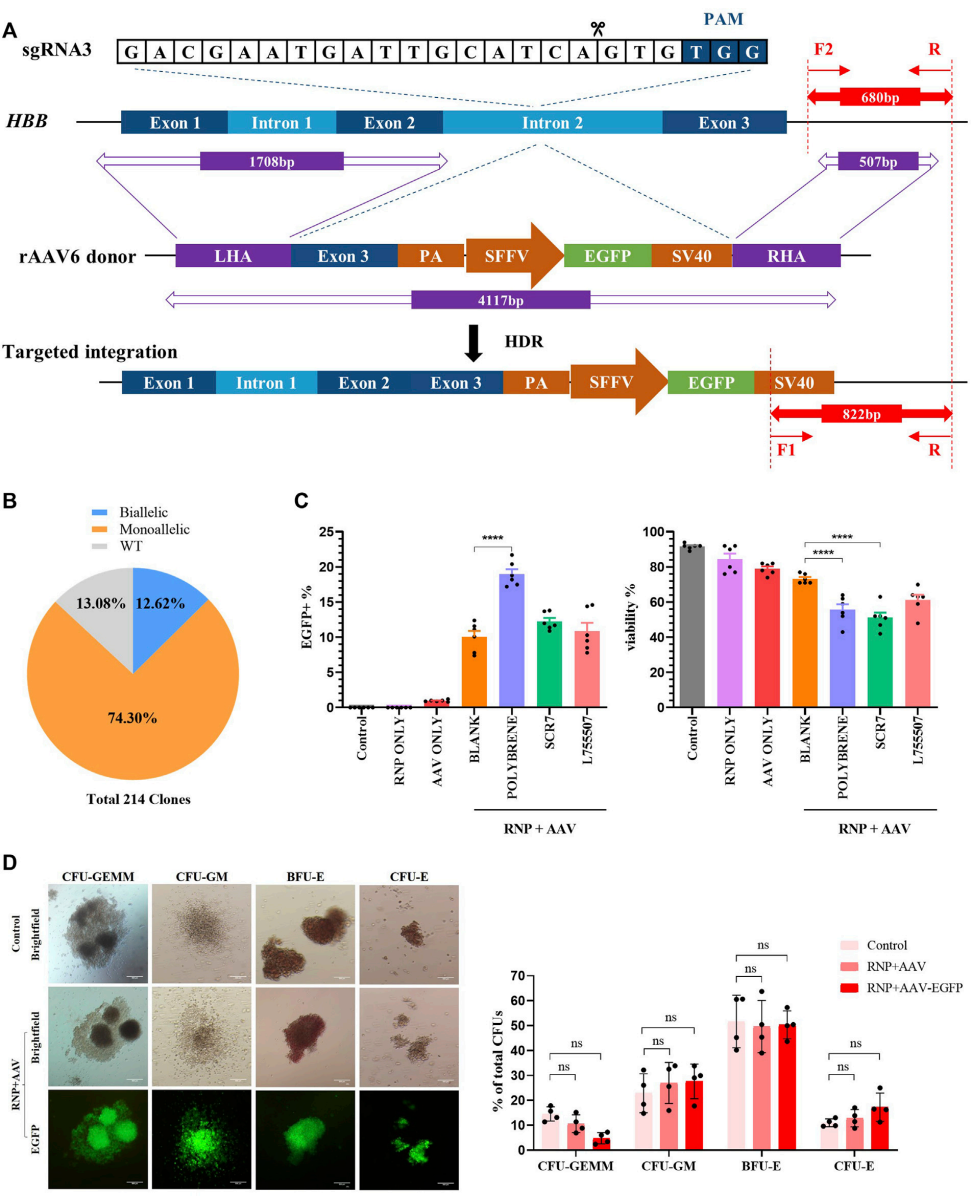
Development of a universal HBB gene correction approach with high HDR efficiency by enrichment of HBB-targeted HSCs. All experiments in this figure were performed on cord blood-derived CD34^+^ cells. **(A)** Schematic overview of the gene targeting strategy for human HBB locus following DSB initiated by Cas9/sgRNA and HDR using the rAAV6 homologous donor as a repair template. Dark blue boxes: HBB exons; light blue boxes: HBB introns; purple boxes: homology arms; green boxes: EGFP selection marker. In–out PCR analysis of the positive colonies with homology-directed repair (HDR) using three primers. **(B)** Pie chart statistics of the colonies’ genotype. A total of 214 colonies were verified. PCR analysis was applied to identify the genotype of methylcellulose colonies from EGFP^+^ HSCs. **(C)** HDR efficiency and viability were analyzed via flow cytometry after HSCs were treated with polybrene and small-molecule compounds such as SCR7 and L755507. **(D)** Representative images of the differentiated HSCs on day 21 showing lineage-restricted progenitors (CFU-GM, BFU-E, and CFU-E) and multipotent progenitors (CFU-GEMM) with EGFP expression. The ratio of different types of colonies was counted. Values are presented as the mean ± SD for quadruplicate samples from a representative experiment. *p*-values were calculated using one-way ANOVA. ** indicates <0.01.

Approximately 60% of the known mutations are located in exons 1 and 2 of the HBB gene. For the purpose of developing a universal strategy to correct a set of hot spot mutations, sgRNA3 was employed to target intron 2 of the HBB locus; site-specific DSB was created by the RNP complex consisting of sgRNA and Cas9 protein; and HDR was achieved using a 4.7 kb rAAV6 homologous donor as a repair template (Figure 2A). An SFFV-EGFP reporter gene was inserted downstream of the HBB gene to facilitate the enrichment of HBB-targeted HSCs. For gene targeting, the Cas9/sgRNA3–RNP complex was electroporated into the cord blood-derived HSCs and transduced with the rAAV6 donor at MOIs ranging from 1 × 10^3^ vgs/cell to 2 × 10^4^ vgs/cell. The results showed that the EGFP^+^ cells reached an average of 17.45% at an MOI of 1 × 10^4^ (Supplementary Figure S2A), and the rates did not increase with higher virus titers. The fluorescence intensity of HSCs reached a plateau at days 4 and 5 after the delivery of RNP and the transduction with rAAV6 (Supplementary Figure S2B). Thus, an MOI of 1 × 10^4^ was chosen for the following experiments.

### Optimizing rAAV6 donor transduction into HSCs to achieve consistently high levels of HDR

After electroporation was conducted with Cas9/gRNA–RNP complexes, the cells were infected with rAAV6 with the treatment of 8 μg/mL polybrene for 18 h, followed by replacement with fresh medium, to enhance the transduction efficiency of rAAV6 to HSCs. Cell viability was estimated using flow cytometry. The results revealed that the rAAV6 transduction efficiency significantly increased in the presence of polybrene but had a detectable effect on cell viability (Figure 2C). Small-molecule compounds, such as SCR7 and L755507, have been reported to significantly enhance CRISPR-mediated HDR efficiency (Maruyama et al., 2015; Liu et al., 2017). The HSCs were cultured in a medium with SCR7 or L755507 at 48 h post-electroporation, and the result showed that the addition of small molecules can slightly enhance AAV transduction efficiency but had no substantial effect on cell viability (Figure 2C).

The EGFP marker can be applied to highly enrich the on-target cells via fluorescent cell sorting. Four days after transfection of Cas9/gRNA–RNP and transduction with rAAV6, the EGFP^+^ population was sorted and cultured in semisolid culture to form various colonies to determine whether the EGFP^+^ HSCs were HBB-targeted cells. “In-out PCR” (primer F2 and primer R amplified the genomic DNA sequence outside the region of the homology arm, and primer F1 and primer R amplified the integrated sequence) was designed to confirm the gene targeting frequencies and allelic distribution in individual colonies. A total of 214 clones were identified, and 86.92% of the clones had targeted integration, with 12.62% containing biallelic integrations and 74.3% containing monoallelic integrations (Figure 2B, Supplementary Figure S2C). This result indicated that the universal HBB gene targeting strategy can achieve high efficiency in HDR-mediated gene editing.

### Functional analysis of CB HPCs after delivery with RNP and the rAAV6 donor

A hematopoietic progenitor CFU assay was conducted to assess whether the *HBB*-targeting strategy could influence the function of HSCs. The sorted EGFP^+^ HSCs can differentiate into all types of blood lineages (CFU-GEMM, CFU-GM, BFU-E, and CFU-E) after plating into the semisolid methylcellulose medium after 14 days of culture. The data demonstrated no significant difference between the control groups and the RNP + AAV-EGFP^+^ groups, and the differentiated colonies consistently expressed high EGFP expression after 14 days of culture (Figure 2D). These findings suggested that the EGFP^+^ population was indeed *HBB*-targeted HSCs. The cell morphology from the *in vitro* erythroid differentiation of HSCs on day 21 was evaluated using Wright–Giemsa staining (Supplementary Figure S2E). Similarly, no differences were observed in the erythroid differentiation ability between the edited and unedited cells (Supplementary Figure S2D), suggesting that editing of HSCs with Cas9/gRNA–RNP combined with rAAV6 does not affect erythropoiesis or the function of HSCs.

### Efficient correction of β-globin mutations by using the universal HBB gene targeting strategy

**FIGURE 3:**
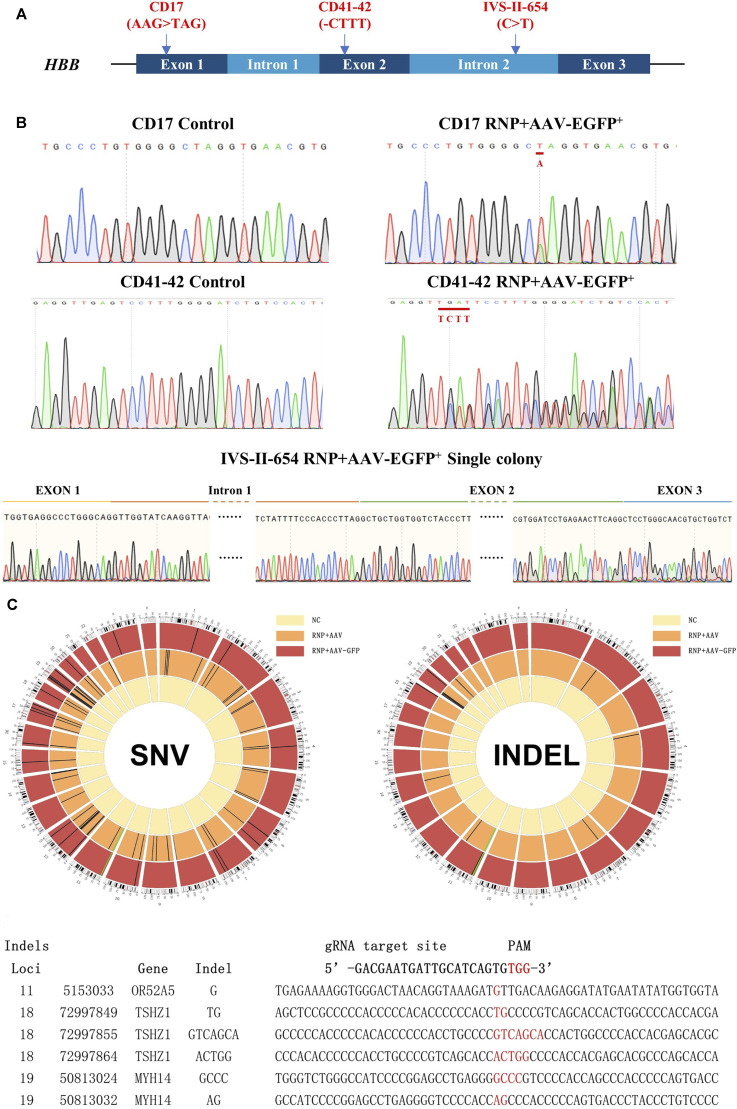
Genetic repair of HBB gene mutations in homozygous β-thal patient-derived HSCs. Three different patient-derived HSCs were used for genetic repair. **(A)** Diagram of universal gene correction of different HBB mutations in patient-derived HSCs. **(B)** Mutation of CD17, CD41-42, and IVS-Ⅱ-654 genetically corrected and confirmed by Sanger sequencing. The repaired locus is indicated by the red line. **(C)** Whole-exome sequencing of gene-untargeted and -targeted HSCs. The black bars represent individual SNVs. Compared with the NC group (untargeted HSCs), the HSCs in the CD41-42 RNP + AAV-GFP group (targeted HSCs) had 23 SNVs, and those in the RNP + AAV group had 72 SNVs. The HSCs in the RNP + AAV-GFP group had six indels, and those in the RNP + AAV group had 22 indels. The idle sequence was detected in the RNP + AAV-GFP group via exome sequencing. No homologous sequences were detected in the indel locus compared with the gRNA3 targeting site.

Next, the strategy was tested to correct the disease-causing mutations in multiple β-thal patient-derived CD34^+^ HSCs (Figure 3A). CD34-positive hematopoietic progenitor cells were acknowledged as capable of self-renewal and multi-lineage differentiation ability and were commonly used for clinical gene therapy for β-thalassemia (Frangoul et al., 2021). Approximately 1,000,000 HSCs were isolated separately from CD41–42 (in exon 2), CD17 (in exon 1), and IVS-II-654 (in intron 2) patient mobilized peripheral blood (mPB) with 98% of CD34^+^ stem/progenitor cells on average (Supplementary Figure S3A). The mPB-derived HSCs were cultured for 2 days to expand, and 1,000,000 HSCs were electroporated with the Cas9/sgRNA3–RNP complex and transduced with an rAAV6 homologous donor. The EGFP^+^ HSCs were sorted and cultured for 4 days, and then the correction of mutation sites was detected by Sanger sequencing. The results revealed remarkable genetic correction efficiency. The AAV6-mediated HDR can achieve averages of 31.99% genetic correction in CD41–42 homozygous patient-derived HSCs and 20.34% in CD17 homozygous patient-derived HSCs (Figure 3B and Supplementary Figure S3B). A colony-forming assay was performed on the corrected β-thal HSCs, and these EGFP^+^ cells can further differentiate into various lineage-restricted colonies after 14 days of culture (Supplementary Figure S3C). The colonies derived from IVS-II-654 gene-targeted HSCs were used for sequencing, and the results confirmed that the HBB gene was successfully corrected in erythroblasts (Figure 3B), consistent with the results of the authors’ previous study on HDR-mediated gene editing in cord blood-derived HSCs.

### Whole-genome sequencing analysis of gene-corrected HSCs

CRISPR/Cas9 technology has highly improved the precise and efficient gene targeting efficiency, but it has the potential risk of causing off-target mutagenesis. Although the potential off-target sites were predicted using CRISPOR and no observable off-targets were confirmed in the top 48 sites through T7E1 assays, high-throughput sequencing was applied to identify the whole exomes of HSCs prior to and after gene targeting to discover rare off-target events. Due to the importance of exon regions, which affect most cell biological functions, this study focused on the analysis of the sequencing results from these regions. The genomic DNA from mixed cells in the untargeted HSC group and the CD41–42 corrected HSC group was detected via whole-genome sequencing. Compared with the untargeted HSCs, the cells in the CD41–42 corrected HSC group were detected to have 23 single-nucleotide variants (SNVs) and six indels (Figure 3C). The number of variations caused by the CRISPR/Cas9 or AAV6 systems was considered. We analyzed the variations, including SNVs and indels, in the CD41–42 corrected HSC group and compared them with the sgRNA3 sequences, and all of them were not within the predicted off-target region. This finding suggested that these variations randomly occurred during cell passaging and were not caused by the gene editing tools.

### Engraftment of gene-edited β-thal patient-derived HSCs in NSI mice

**FIGURE 4:**
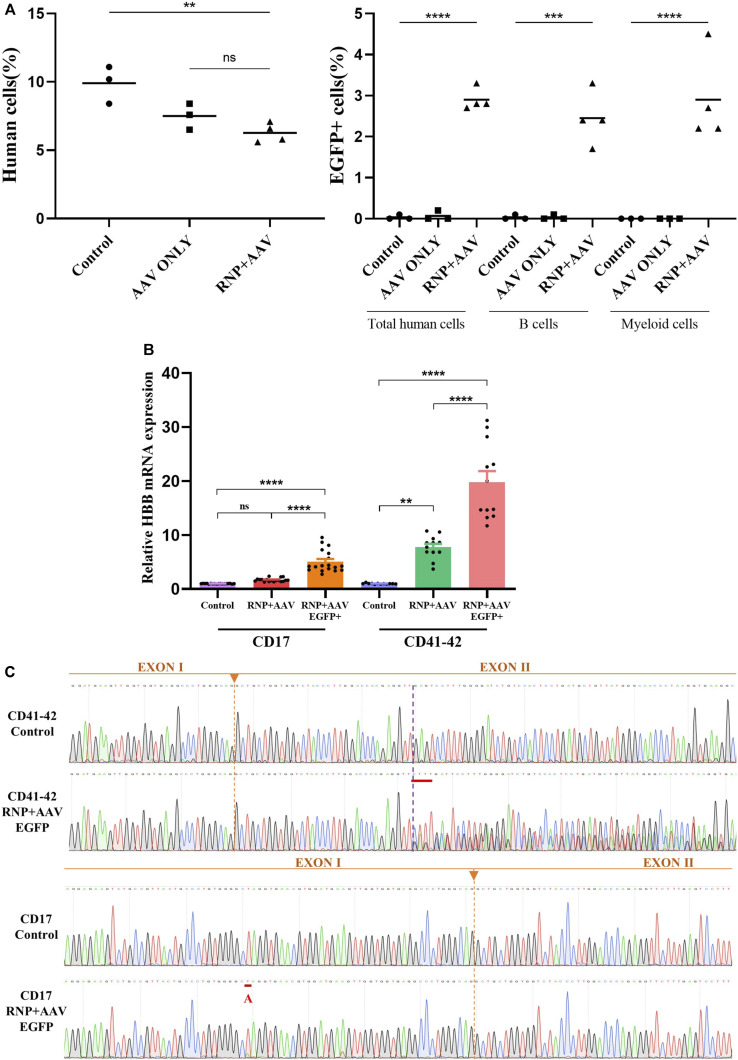
Restoration of HBB transcription after gene correction and an *in vivo* engraftment capability assay **(A)** Mouse bone marrow was analyzed via flow cytometry for human cell engraftment 16 weeks post-transplantation. **(B)** HBB mRNA expression quantified by RT-qPCR in erythrocytes differentiated from HSCs in the control, RNP + AAV, and RNP + AAV EGFP^+^ groups. The relative gene expression levels were normalized by the housekeeper gene GAPDH. All data are represented as the mean ± SD. *p < 0.05 and **p < 0.01 by the Student’s t-test. **(C)** Confirmation that the HBB gene was successfully corrected in erythroblasts derived from patient gene-corrected HSCs by Sanger sequencing.

The HSCs derived from CD41–42 patients mobilized peripheral blood HSCs were used to determine whether the gene-corrected HSCs retained the engraftment ability because of their high clinical relevance. Four days after the electroporation of the Cas9/sgRNA–RNP complex and AAV6 donor delivery, an equivalent cell dose of 10^6^ viable CD41–42 corrected EGFP^+^ HSCs was transplanted into NSI mice by tail-vein injection. After 16 weeks post transplantation, the mice were euthanized and BM was harvested to determine the implantation rate. The data in the Control and AAV ONLY groups were derived from three transplanted mice, and the data in the RNP + AAV group were obtained from four transplanted NSI mice. All groups of transplanted mice displayed human engraftment in the BM, as evidenced by flow detection in the presence of hCD45^+^/HLA-ABC double-positive cells (Supplementary Figure S4A). Compared with the Control group, the treatment groups showed a decrease in the rates of human cell chimerism, and the AAV ONLY and RNP + AAV groups displayed similar chimerism rates (Figure 4A). The percentage of EGFP expression in human cell chimerism was 2.9%. In addition, the myeloid (CD33) and lymphoid (CD19) reconstitution had averages of 2.9% and 2.45% EGFP^+^ cells, respectively (Figure 4A). Collectively, these results confirmed that the gene-targeted HSCs have engraftment potential and lineage reconstitution ability *in vivo*.

### Transcription of HBB restoration after gene correction

The CD41–42 and CD17 patient-derived HSCs were subjected to a 21-day *in vitro* erythroid differentiation prior to and after gene correction to determine whether the gene correction of patient-derived HSCs could restore the expression of full-length β-globin. Flow cytometric analyses showed a high proportion of CD71^+^/CD235a^+^ cells, indicating the successful differentiation of HSC-derived erythroblasts (Supplementary Figure S4B). Conventional RT-PCR was applied to amplify the HBB cDNA from the erythroblasts, and the results showed that the level of β globin significantly increased in gene-corrected erythroblasts compared with its un-corrected counterpart (Figure 4B). Sanger sequencing also confirmed the HBB cDNA was successfully restored after gene correction in patient-derived HSCs and erythroblasts (Figure 4C).

## Discussion

The technologies of CRISPR/Cas9-based gene correction are currently being developed as a powerful tool to cure a wide range of congenital blood diseases, including β-hemoglobinopathies, such as SCD and β-thal; severe combined immunodeficiency diseases (Schiroli et al., 2017); and other metabolic diseases (Gomez-Ospina et al., 2019). Previous studies mainly focused on the gene correction of SCD with a single point mutation (A > T) in the sixth codon of the β-globin gene. However, unlike SCD, β-thal represents more than 200 different types of mutants in the HBB gene with distinct phenotypes. In the present study, a comprehensive curative strategy was established to correct most of the 100 types of HBB mutations by using validated CRISPR gRNAs and one donor DNA template. The strategy could have an advantage for future clinical applications using patient-derived stem cells. Due to the limited DNA payload of rAAV6 (<4.7 kb) and the purpose of including a wide range of mutations, a gRNA targeting intron 2 of the HBB gene in combination with the rAAV6 donor was designed to achieve HDR. The homologous arms span 4.1 Kb of the HBB locus containing not only the upstream mutations of the HBB gene (such as −28) but also the downstream mutations in exons and introns found in patients with β-thal, different from the cDNA donor used in the common treatment strategy. The EGFP gene was introduced as a selection marker to enrich the gene-targeted HSCs and evaluate the targeting efficiency by EGFP percentages. Notably, the gene correction efficiency was remarkably high, up to 86.92%. Although the off-target effect has been frequently reported in CRISPR/Cas9-mediated cell lines, mice, and other species (Fu et al., 2013; Naeem et al., 2020), we did not detect off-target mutations caused by endonucleases in corrected HSCs through silico predictions and whole-genome sequencing, demonstrating that the proposed strategy is safe, which is crucial for clinical applications.

Multiple methods were designed to optimize the universal HBB gene targeting strategy to achieve high HDR efficiency in HSCs. For gene targeting, the CRISPR/Cas9 complex can be delivered in the forms of plasmids, mRNA (IVT sgRNA), or RNP (Cas9 protein complexed with sgRNA). The three forms were compared, and the results showed that the modified synthetic sgRNA was significantly higher than the IVT sgRNA or plasmid in terms of indel ratio and cell viability. Considering that RNPs cleave chromosomal DNA very quickly after delivery and are degraded rapidly in cells, RNP delivery has advantages of higher genome-editing efficiency and lower off-target effect over the Cas9 plasmid or mRNA. Thus, RNP delivery via electroporation has emerged as a powerful strategy for genome editing in several mammals. Historically, achieving HDR in human HSCs was difficult because of the low delivery efficiency of DNA donors. In this study, rAAV6 donor transduction was optimized by titrating the MOI or testing small-molecule compounds, such as Scr7 and L755507, which greatly enhanced the HDR rates in HSCs, with an appropriate cell survival rate. Scr7 is a specific DNA ligase IV inhibitor that can improve HDR efficiency by inhibiting the nonhomologous end-joining pathway (Vartak et al., 2018), and L-755507 is a selective β3 adrenergic receptor partial agonist that enhances the HDR efficiency (Chen et al., 2022). These two small-molecule compounds have been proven to improve the HDR ratio in mammalian cells, mouse embryonic stem cells, and induced pluripotent stem cells in other studies. Scr7 and L755507 can also slightly increase the HDR efficiency depending on the concentration of the compounds. Polybrene significantly enhances gene-targeting efficiency in HSCs. However, it has a significant effect on cell viability, which is not conducive to subsequent functional studies and NSI mouse transplantation experiments.

The universal *in situ* HBB gene-corrected strategy proposed in this study could be adapted to repair various HBB gene mutations in patient-derived HSCs, which is important for clinical translation. CD34^+^ HSCs derived from CD41–42, CD17, and IVS-II-654 patients with β-thal were edited with Cas9/sgRNA–RNP complexes and AAV6 donor delivery. By using this donor, averages of 20.34% CD17 homozygous patient-derived HSCs and 31.99% CD41-42 homozygous patient-derived HSCs were targeted, and the seamless integration of exons 2 and 3 was confirmed by Sanger sequencing in IVS-II-654 patient-derived HSCs. According to a recently published clinical study, two patients—one with transfusion-dependent β-thalassemia (TDT) and the other with SCD—received autologous CD34^+^ cells edited with CRISPR/Cas9 targeting the BCL11A enhancer. The TDT patient underwent myeloablative conditioning and was infused with CTX001 (16.6 × 10^6^CD34^+^ cells per kilogram) on day 1. The knockout frequency of allelic editing was approximately 80% and her hemoglobin level normalized to 12.1 g per deciliter at month 4 and remained normal through month 18 without transfusion. By that analogy, the method reported in our study would require at least 41.5 × 10^6^ CD34^+^ of gene-corrected HSCs by HDR for the gene therapy application. The restoration in the transcription of the HBB gene was confirmed via RT-qPCR and Sanger sequencing in red blood cells differentiated from the gene-corrected HSCs. Finally, the results demonstrated that the gene-targeted HSCs were able to engraft in NSI mice. However, a reduction was observed in the percentage of chimeric cells at 12 weeks post-transplantation in the RNP + AAV group compared with the unedited cell groups, which may be because the gene-modified HSCs were deficient in the ability of cell expansion and differentiation, which was consistent with the results reported in the previous article (Bak et al., 2018). Further development, such as the exploitation of *ex vivo* HSC culture and expansion media and the identification of small-molecule drugs that can effectively enhance the HDR efficiency of HSCs (Sakurai et al., 2023), is needed to help solve these problems and make the approach reach clinical application.

In conclusion, this study provides efficient and universal genome editing therapies not only for β-thal but also for a range of other hematological diseases associated with disease-causing mutants in patient-specific HSCs. Although EGFP was used as a selection marker to highly enrich the transfected cells, which is not suitable for clinical use, other similar signaling-inert cell surface markers, such as the truncated nerve growth factor receptor (tNGFR), could be applied to enrich the gene-corrected HSCs to increase the feasibility of clinical applications in further studies.

## Data Availability

The datasets presented in this study can be found in online repositories. The names of the repository/repositories and accession number(s) can be found at: NCBI, PRJNA11014982.

## Abbreviations

CFU, colony-forming unit; DSB, double-strand break; FACS: fluorescent-activated cell sorting; HDR, homology-directed repair; HSCs, hematopoietic stem cells; RNP, ribonucleoprotein complex; rAAV6, recombinant adeno-associated virus 6; sgRNA, small-guide RNA; ssODNs, single-strand oligodeoxynucleotides; tNGFR, truncated nerve growth factor receptor.
