# Supplementary material for: In situ correction of various β-thalassemia mutations in human hematopoietic stem cells
Source: Front Cell Dev Biol. 2024 Jan 25;11:1276890. doi: 10.3389/fcell.2023.1276890 (PMC10850376; doi:10.3389/fcell.2023.1276890)
Supplement: Supplementary file 1 [file DataSheet1.PDF]

Figure S1

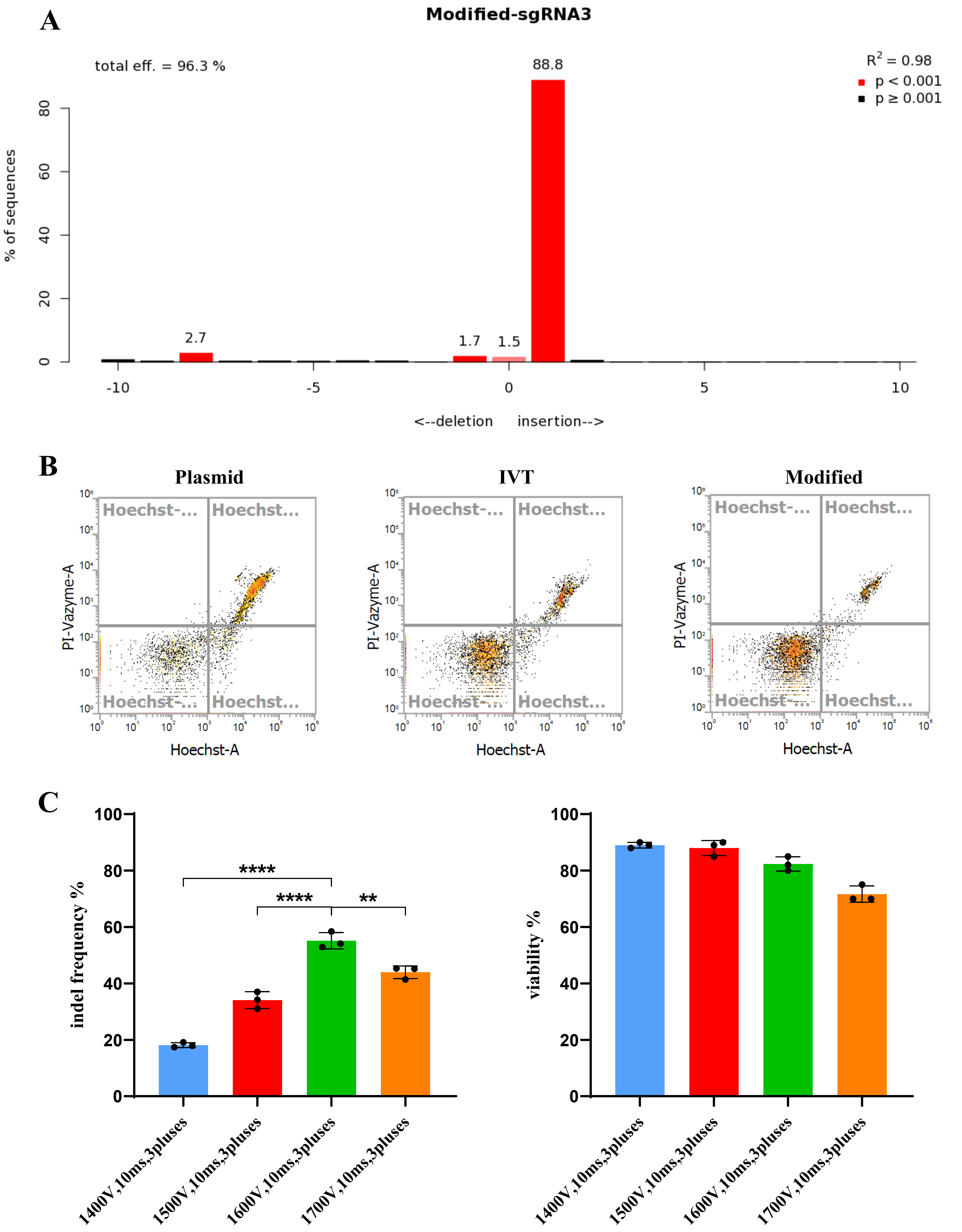
A
Modified-sgRNA3
total eff. = 96.3 %
88.8
R2 = 0.98
p < 0.001
p ≥ 0.001
% of sequences
2.7
1.7
1.5
<--deletion
insertion-->
B
Plasmid
IVT
Modified
PI-Vazyme-A
Hoechst-A
C
indel frequency %
****
****
**
viability %
1400V,10ms,3pluses
1500V,10ms,3pluses
1600V,10ms,3pluses
1700V,10ms,3pluses

Figure S1. (A) The indel frequency of Cas9/sgRNA3 targeted to the *HBB* intron locus in pools of HSCs was assessed by TIDE software. (B) Flow cytometric analysis of the survival rate of HSCs electroporated with different sgRNA treatments, in plasmid, in vitro transcription (IVT) or modified sgRNA. (C) The indel frequency and cell viability of different electroporation parameters assessed by TIDE software and flow cytometric after electroporation in HSCs.

Figure S2

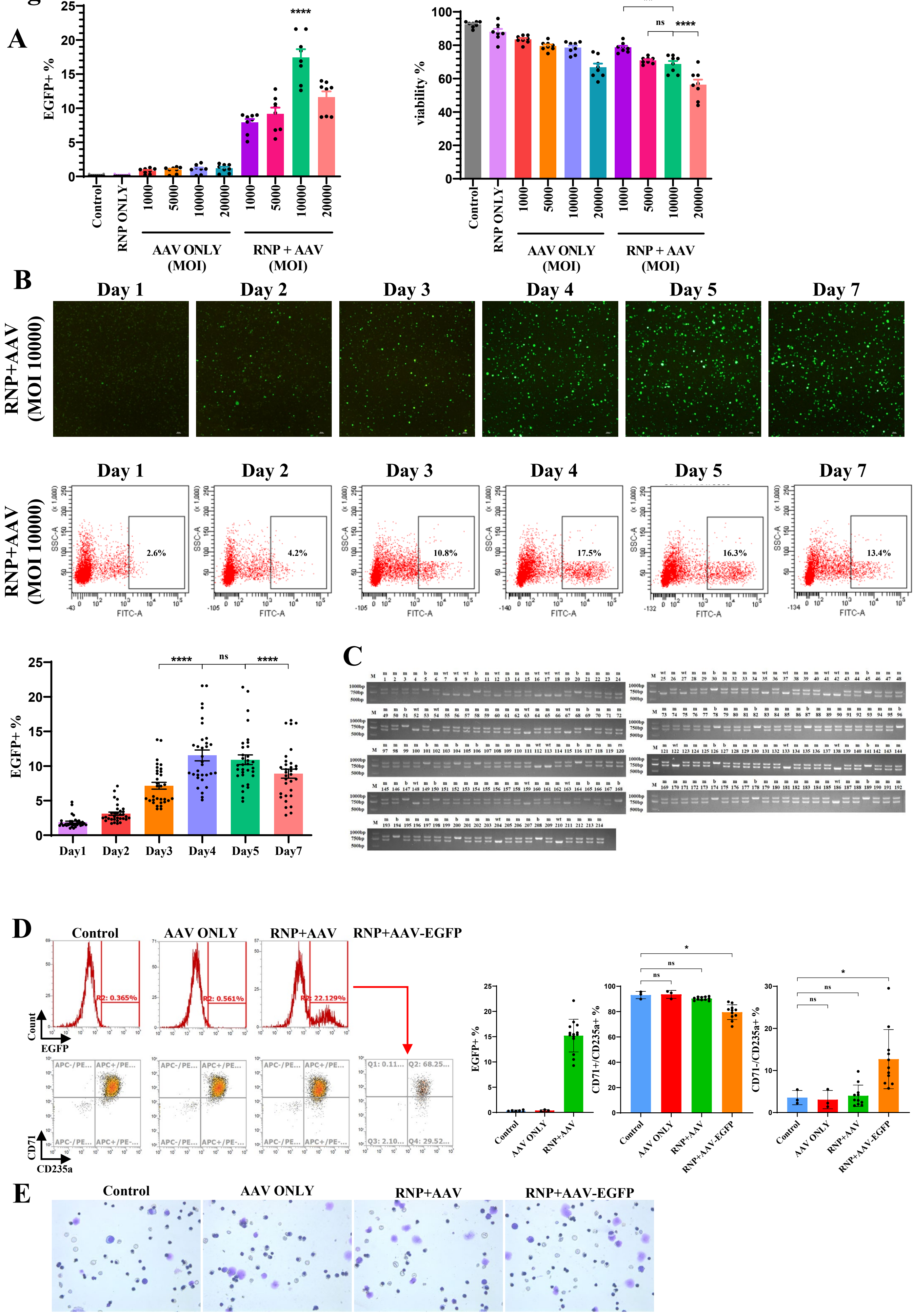

A
EGFP+ %
****
Control
RNP ONLY
1000
5000
10000
20000
AAV ONLY (MOI)
RNP + AAV (MOI)
viability %
**
ns
****
B
RNP+AAV (MOI 10000)
Day 1
Day 2
Day 3
Day 4
Day 5
Day 7
SSC-A (x 1,000)
FITC-A
2.6%
4.2%
10.8%
17.5%
16.3%
13.4%
EGFP+ %
Day1
Day2
Day3
Day4
Day5
Day7
C
M
1000bp
750bp
500bp
D
Control
AAV ONLY
RNP+AAV
RNP+AAV-EGFP
Count
EGFP
R2: 0.365%
R2: 0.561%
R2: 22.129%
CD71
CD235a
Q1: 0.11...
Q2: 68.25...
Q3: 2.10...
Q4: 29.52...
EGFP+ %
CD71+/CD235a+ %
CD71-/CD235a+ %
*
ns
E
Control
AAV ONLY
RNP+AAV
RNP+AAV-EGFP

Figure S2. (A) The EGFP efficiency and cell viability of different rAAV6 MOIs was analyzed by flow cytometry 4 days after the delivery of RNP and rAAV6 into HSCs. (B) The fluorescence intensity of HSCs transduced with the rAAV6 from Day1 to Day7, the rAAV6 MOI was 10^4. All data represent mean ± SD. **P<0.01 by one-way ANOVA test. (C) PCR analysis was applied to identify the genotype of methylcellulose colonies from $EGFP^+$ HSCs.(D) Flow cytometric analysis of erythrocytes from Control, AAV only, RNP+AAV and RNP+AAV-EGFP groups using the surface markers CD71 and CD235a at day21. (E)Wright-Giemsa staining of the cultured erythroid differentiated from HSCs at day21 in the Control, AAV only, RNP+AAV and RNP+AAV-EGFP groups.

Figure S3

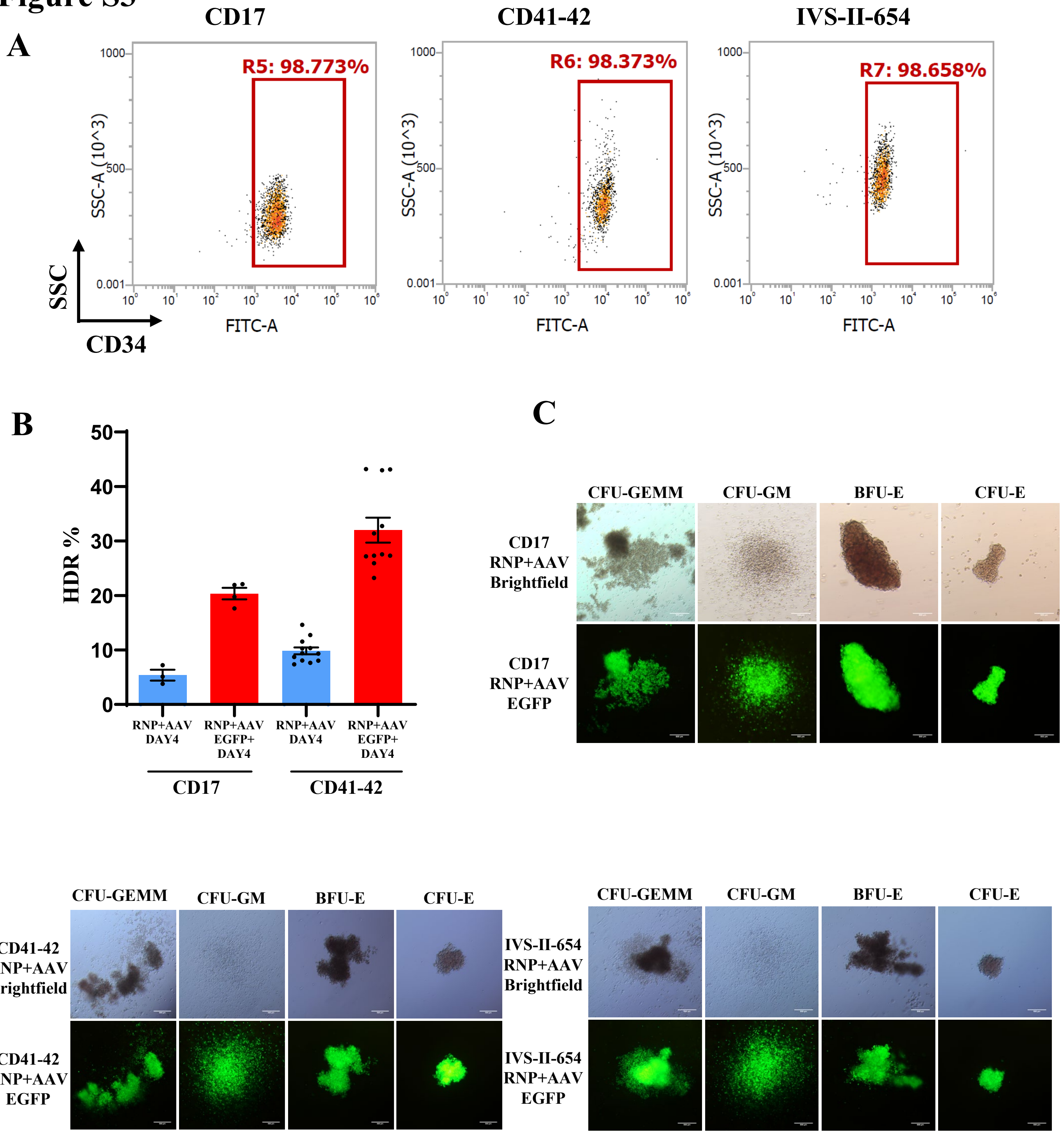

Figure S3. (A) Flow cytometric analyses of hematopoietic stem cells ($CD34^+$) isolated from the CD41-42、CD17 and IVS-II-654 patients mobilized peripheral blood. (B) Collective data from the analysis of $EGFP^+$ cells and HDR efficiency in the Control and RNP+AAV groups from CD41-42 and CD17 patients HSCs. (C) Representative images of colony formation units (CFUs) derived from the gene corrected β-Thal HSCs with high level of EGFP expression.

Figure S4

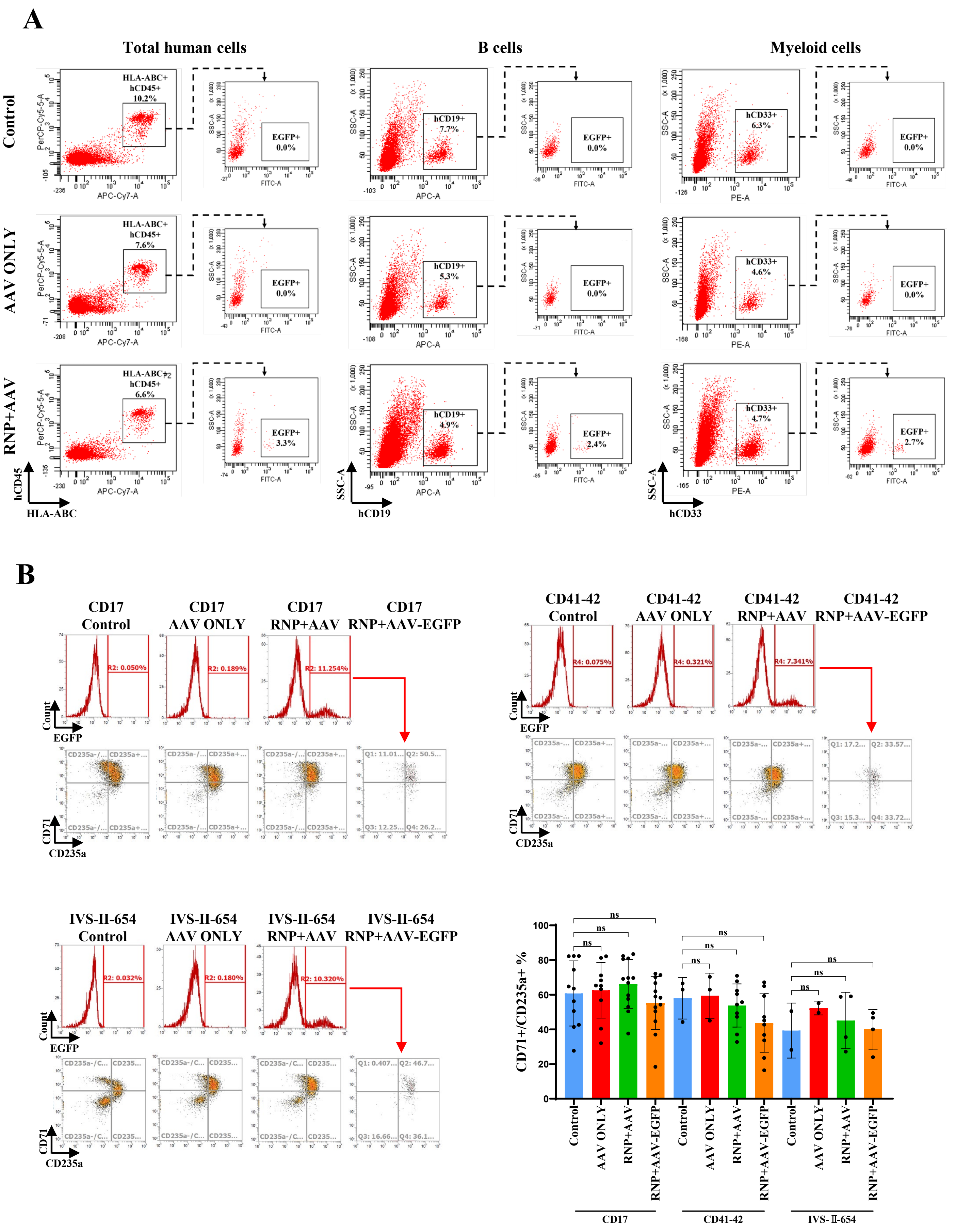

A
Total human cells
B cells
Myeloid cells
Control
AAV ONLY
RNP+AAV
HLA-ABC+
hCD45+
10.2%
7.6%
6.6%
EGFP+
0.0%
3.3%
hCD19+
7.7%
5.3%
4.9%
2.4%
hCD33+
6.3%
4.6%
4.7%
2.7%
hCD45
HLA-ABC
SSC-A
hCD19
hCD33
B
CD17
Control
AAV ONLY
RNP+AAV
RNP+AAV-EGFP
R2: 0.050%
R2: 0.189%
R2: 11.254%
CD41-42
R4: 0.075%
R4: 0.321%
R4: 7.341%
IVS-II-654
R2: 0.032%
R2: 0.180%
R2: 10.320%
Count
EGFP
CD71
CD235a
CD71+/CD235a+ %
ns

Figure S4. (A) Mouse bone marrow was analyzed by flow cytometry for human cell engraftment 16 weeks post-transplantation. (B) Representative FACS plots from Day 21 of differentiation show cell surface markers associated with erythrocytes ($CD71^{+}/CD235a^{+}$) from the gene corrected β-Thal HSCs.
